# The value of intraoperative parathyroid hormone assay in the surgery of mediastinal ectopic parathyroid adenoma (A case series)

**DOI:** 10.1016/j.amsu.2019.06.012

**Published:** 2019-06-27

**Authors:** Sani Rabiou, Sani M. Aminou, Boubacar Efared, Hicham Harmouchi, Marwane Lakranbi, Mounia Serraj, Yassine Ouadnouni, Mohamed Smahi

**Affiliations:** a Department of Thoracic Surgery, CHU Hassan II, Fes, Morocco; b Department of Diabetology, Endocrinology Ant Metabolism Disorder, Niamey National Hospital, Niger; c Department of Pathology, CHU Hassan II, Fes, Morocco; d Department of Pneumology and Respiratory Disorder, CHU Hassan II, Fes, Morocco; e Faculty of Medicine, Abdou Moumouni University, Niamey, Niger; f Faculty of Medicine and Pharmacy, Sidi Mohamed Ben Abdellah University, Fez, Morocco

**Keywords:** Hyperparathyroidism, Parathyroid adenoma, Malignant hypercalcemia, Scintigraphy, Surgery, Postoperative hypocalcaemia, MRI, magnetic resonance imaging, CT, computed tomography

## Abstract

**Background and aims:**

Ectopic parathyroid adenoma is a rare entity. Its clinical management is challenging due to varying locations in the mediastinum. The aim of our study is to report our experience about the preoperative localization of the ectopic parathyroid adenoma and to emphasize the major role of the intraoperative parathyroid hormone assay in such circumstances.

**Methods:**

It is a monocentric, retrospective study about patients diagnosed with EPA (ectopic parathyroid adenoma) from January 2015 to December 2016. Clinical aspects, preoperative management as well as the surgical procedures have been analyzed.

**Results:**

There were 7 women, with an average age of 59.14 years. Six patients presented with biological disorders of the phosphocalcic metabolism such as spontaneous bone fracture and recurrent renal lithiasis. In one case, EPA was discovered in the setting of malignant hypercalcemia. The topographic preoperative assessment with a cervicothoracic CT (computed tomography) showed spontaneously hyperdense tissular masses of variable localizations in the mediastinum. A Tc-99 m (99mTc - MIBI) scintigraphy was performed in 5 patients and showed uptake in all cases. We performed cervicotomy in 1 case, manubriotomy in 2 patients, neck manubriotomy in 2 cases, total vertical sternotomy in 1 case, and posterolateral thoracotomy in 1 patient. The lesion was localized in the mediastinum in 1 patient in the perithymic fat in 1 case; EPA was laterotracheal in 1 case, retro tracheal in 1 case, intra-thymic in 2 cases, inter-jugulo-carotidian with contact with the left subclavial artery in 1 case, and anterior mediastinal in 1 patient. The 1-h after-parathormonemia following removal of the surgical specimens showed a decrease value of 45 and 80% of the baseline value. No surgical morbidity was noted after an average follow-up of 7.9 months (range of 5–18 months).

**Conclusion:**

The preoperative topographic diagnosis of ectopic parathyroid adenoma is challenging for the surgeon despite progress in the morphological assessment. The intraoperative parathyroid hormone assay is a valuable tool for an appropriate surgical management.

## Highlights

• Thoracic surgeons faced difficulties in the management of ectopic parathyroid adenomas in the mediastinal location.
• The intraoperative assay of parathormonemia helps guide the surgeon in his strategy of complete excision of the ectopic parathyroid adenom
• Hypocalcemia is a constant postoperative complication and its absence may make questionnable the quality of the tumor resection.

## 1 Introduction

The ectopic parathyroid tissue is the cause of most frequently reported failure in surgery of hyperparathyroidism, resulting mostly in reoperation [1]. Congenital or acquired, its topographic preoperative diagnosis remains difficult despite advances in exploration techniques that become more and more sophisticated [2]. Thus, intraoperative diagnosis must be guided by a surgical dissection strategy to better identify the gland. The aim of our study was to evaluate our strategy in preoperative localization of the ectopic parathyroid adenoma, emphasizing the contribution of the intraoperative parathormone assay in this context.

## 2 Materials and methods

We conducted a single-center retrospective study on patients managed for ectopic parathyroid adenoma during the period extending from January 2015 to December 2016. For the management of these patients, our strategy was to systematically perform the serum parathormone assay during the surgery. The assay is carried out in collaboration with the anaesthesiologists team 1 h after the removal of the parathyroid tissue. The patient's surgical closure was performed only after having obtained a decrease of parathormonemia at least 50% compared to the initial rate. We retrospectively recorded 7 patients admitted for surgical management of a parathyroid adenoma with mediastinal location. The age, sex, the circumstances of discovery, the preoperative evaluation, the choice of surgical approach, the surgical procedure and the postoperative evolution of patients were reported on a survey sheet and then analyzed. The work has been reported in line with the STROCSS criteria [3].

## 3 Results

**Fig. 1:**
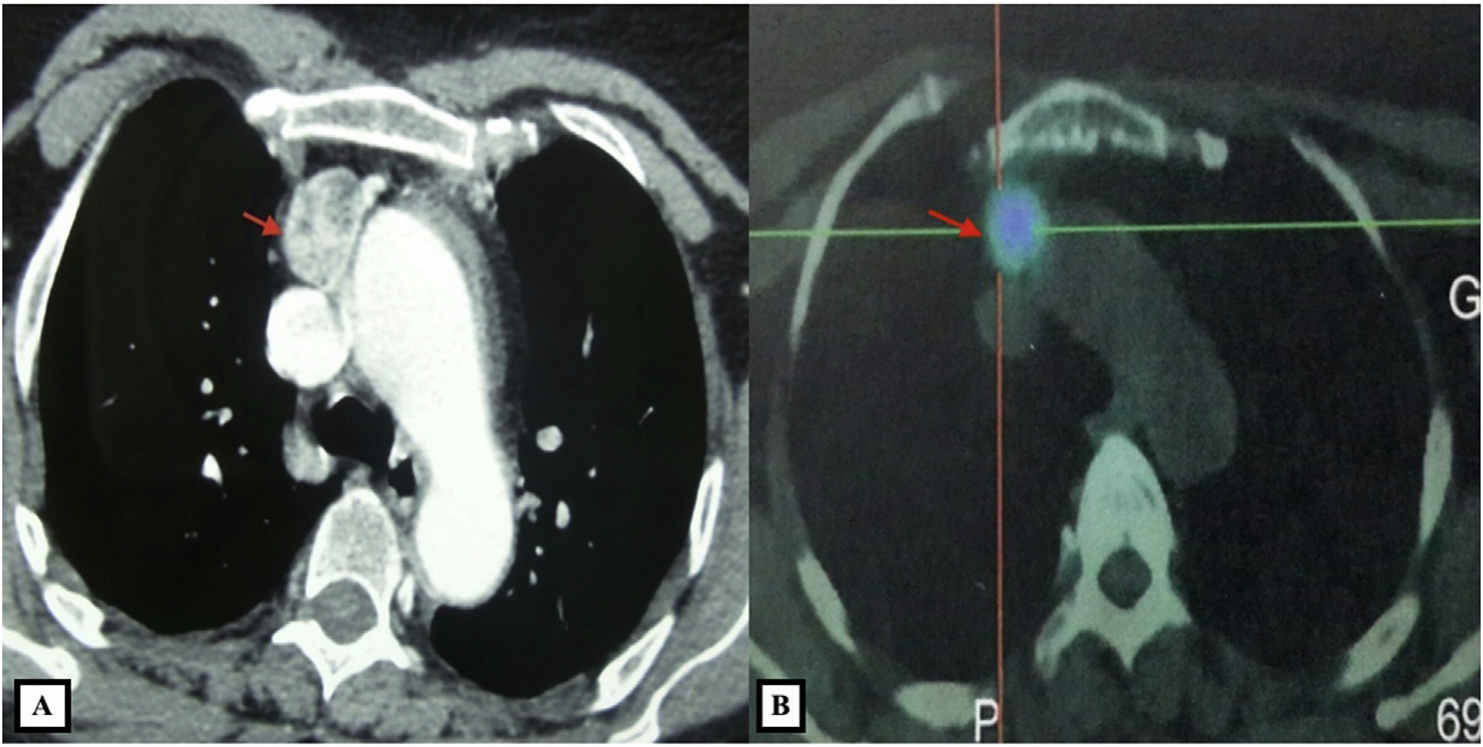
**A:** Thoracic CT-scan showing anterior mediastinal tissular mass with enhancement after contrast injection. **B:** Sestamibi-scan (99 m Tc – MIBI) showing intense uptake by the tissular mass.

**Fig. 2:**
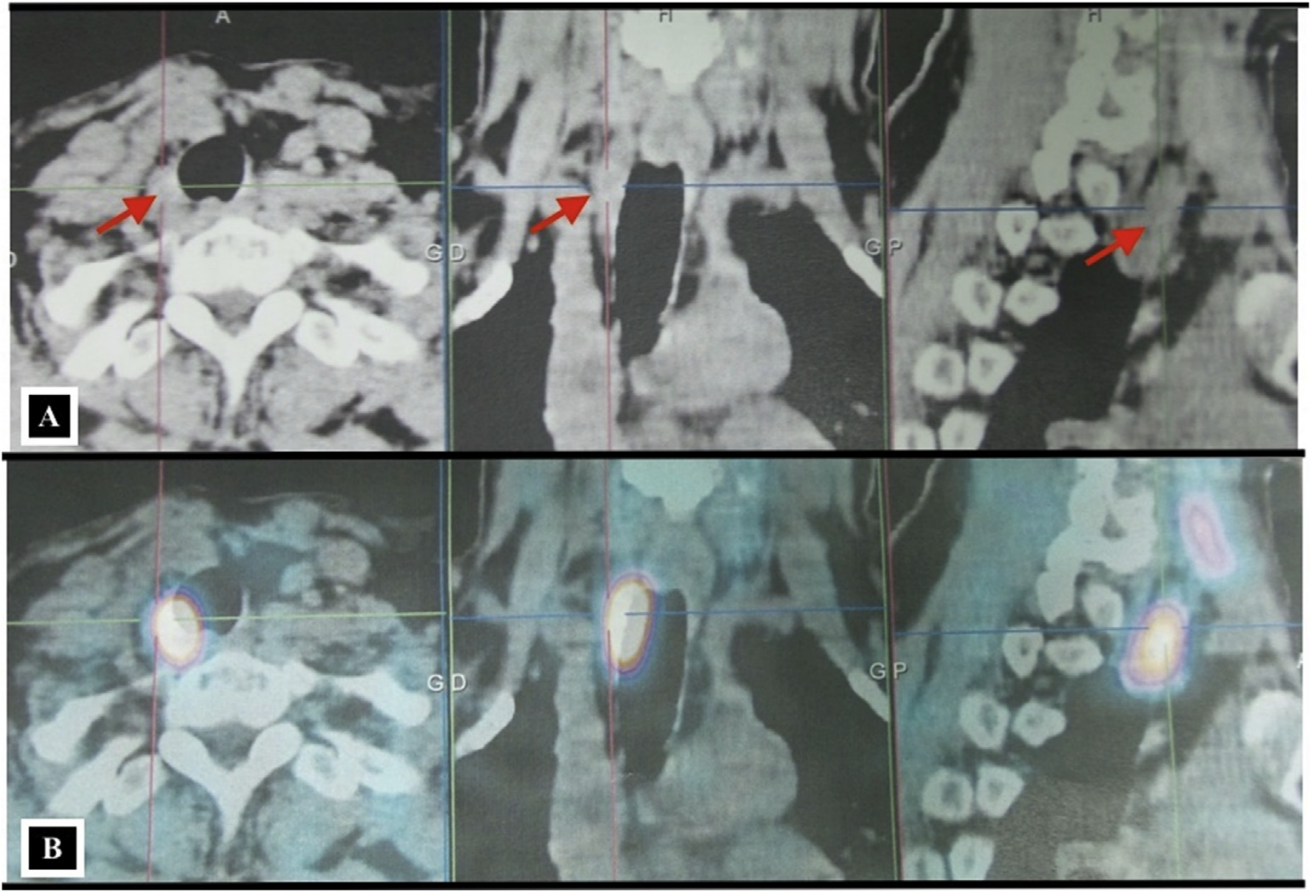
**A:** Cervico-thoracic CT-scan showing a right latero-tracheal mass that is spontaneously hyperdense. **B:** On sestamibi-scan (99 m Tc – MIBI) this mass shows an important tracer uptake.

**Fig. 3:**
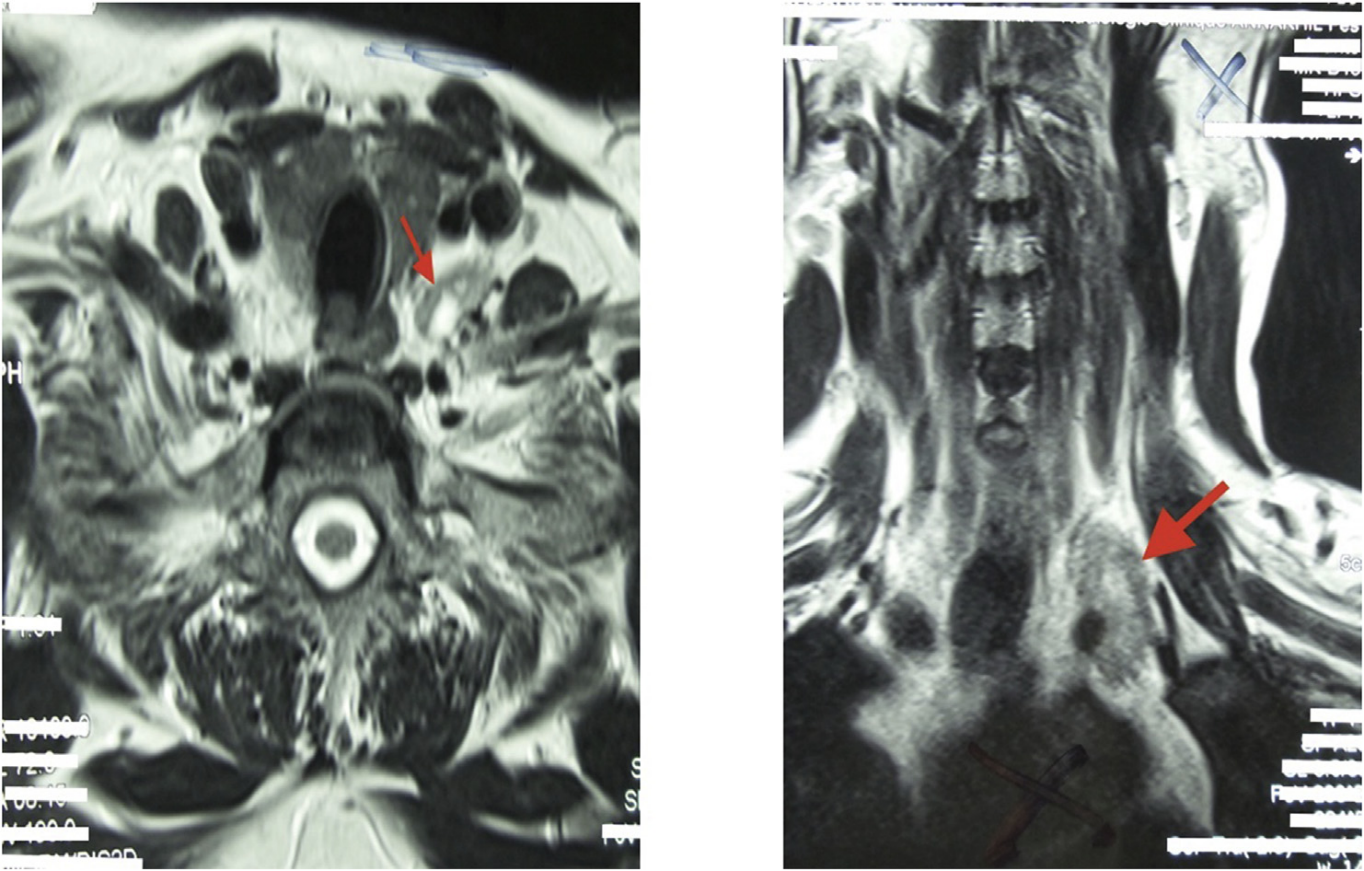
CT-scan showing interjugulo-carotidian mass corresponding to a parathyroid adenoma.

**Fig. 4:**
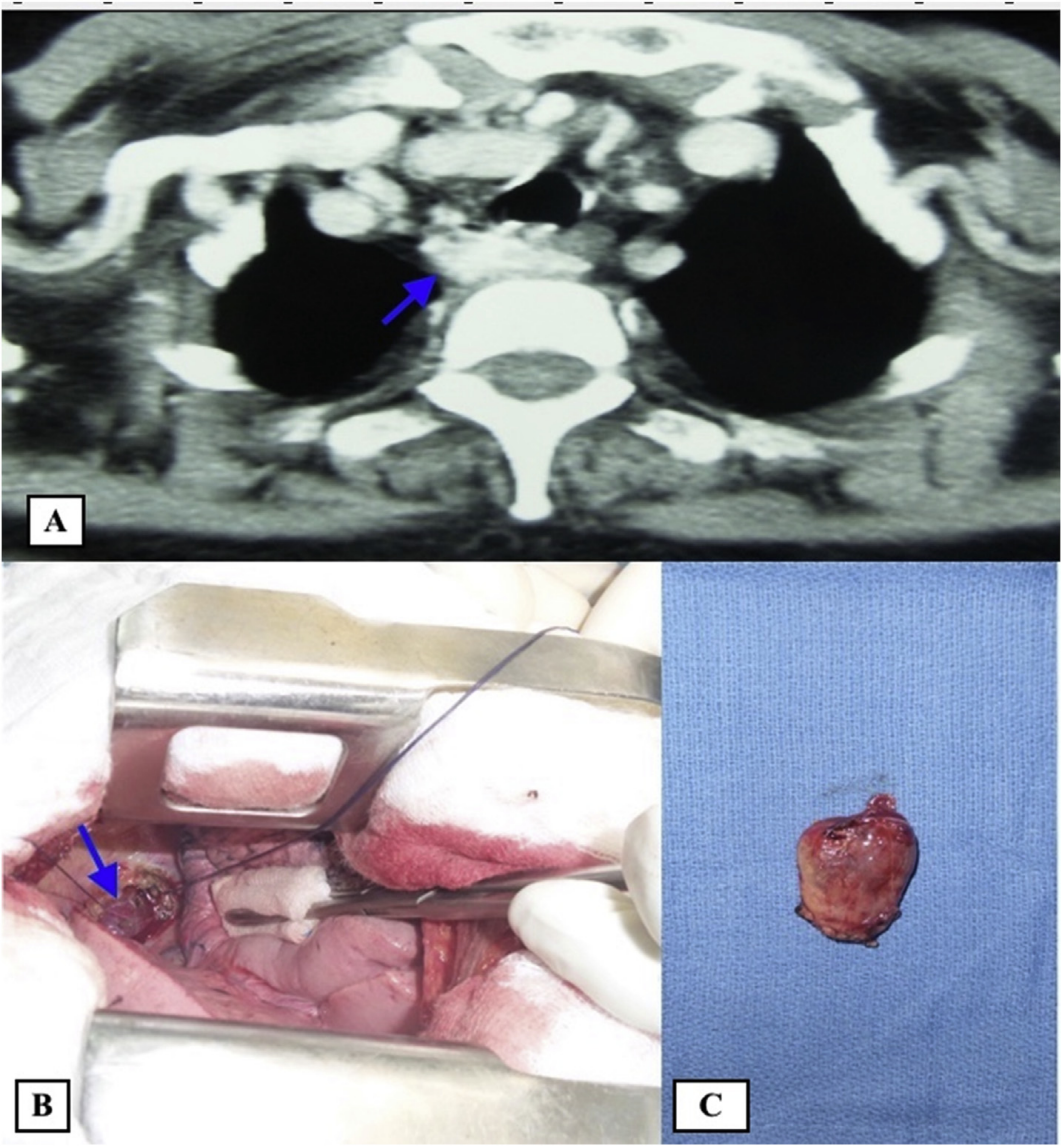
**A:** Thoracic CT-scan showing a retrotracheal mass with enhancement after contrast injection. **B:** Intraoperative view of the tissular mass described above. **C:** Macroscopic view of the lesion after surgical excision.

**Fig. 5:**
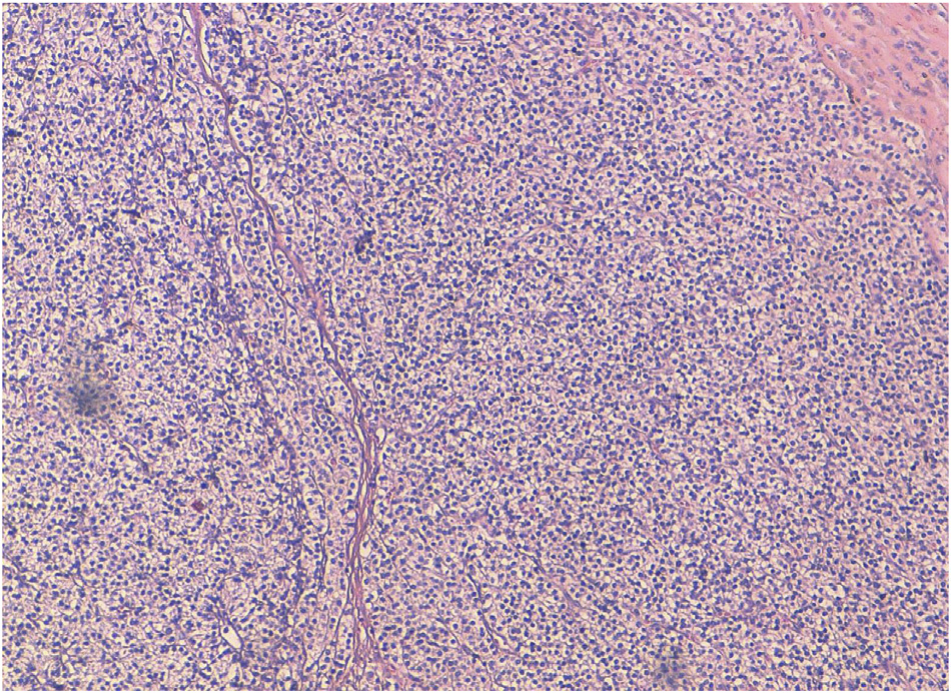
Histological view of a case of parathyroid adenoma showing admixture of chief cells, oxyphilic cells and clear cells, arranged in sheets and cords (Hematoxylin & Eosin x 50).

**Fig. 6:**
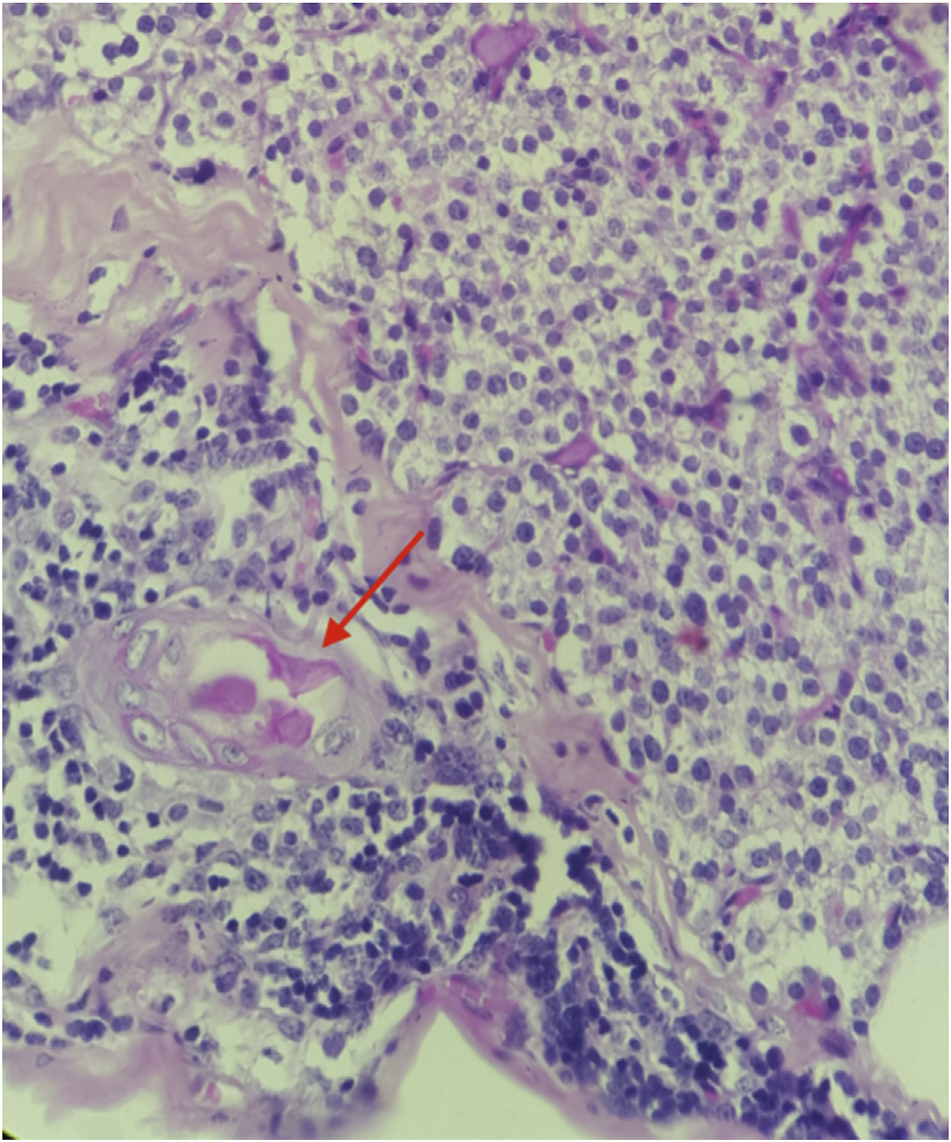
Histological view of a case of intrathymic parathyroid adenoma. Thymic parenchyma with Hassall's corpuscle is shown here at the left part of the image (arrow) (Hematoxylin & Eosin x 50).

There were 7 women aged of 44–70 years with an average age of 59 years. Three patients were followed for kidney stones. The mode of discovery was a spontaneous fracture of the femur in 2 patients and spine in another patient. The discovery was fortuitous in 1 patient during a work-up in the setting of malignant hypercalcemia. Table 1 summarizes the results of laboratory tests showing hypercalcemia and hyperparathyroidism. On topographical evaluation, cervical CT showed a tissue mass, spontaneously hyperdense with variable location in the mediastinum according to patients (Fig. 1, Fig. 2, Fig. 4A). The study of its relationship with the adjacent mediastinal structures was the rule after injection of iodinated contrast material. A magnetic resonance imaging (MRI) was performed in one case in a patient already operated for parathyroid adenoma by cervicotomy with failure (Fig. 3). Five patients underwent scintigraphy with technetium 99 (99mTc - MIBI) to better characterize the parathyroid nature of the tissue. In these 5 patients, we noted an uptake in lesions already described on CT (Fig. 1, Fig. 2B). In the other 2 patients, scintigraphy with technetium-99 (99mTc - MIBI) was not made because of a technical malfunction at the nuclear medicine department, which is the only center covering the whole region. The choice of surgical approach was made based on theoretical difficulties of dissection, predictable on the morphological assessment and depending on the location of the lesion in the mediastinum. We have performed a cervicotomy in 1 case, a manubriotomy and neck manubriotomy in 2 cases, a total vertical sternotomy in 1 case, and posterolateral thoracotomy in 1 patient. Surgical exploration helped to locate the lesion in the mediastinum, which was laterotrachial (1 case), retrotracheal (1 case), intrathymic (2 cases), inter-jugular carotidian with contact with the left subclavian artery (1 case), in the fat of the thymic compartment (1 case), anterior mediastinal between the superior vena cava and the artery brachiocephalic (1 case) (Fig. 4B and C). To minimize the risk of failure and reoperation, we realized systematically an assay of parathyroid hormone an hour after the removal of the specimen. The intraoperative monitoring was considered satisfactory if there was a decrease of parathormonemia of at least 50% compared to the initial rate. We noted a variable decrease in parathyroid hormone between 45 and 80% compared to baseline levels. So dissection time and operative time were relatively longer in patients with intraoperative parathyroid hormone decrease of 45% compared to the others. Pathological examination of all resected parathyroid specimens measured between 1.5 and 3.7 cm. The final histological diagnosis was parathyroid adenoma for all 7 cases (Fig. 5, Fig. 6). The postoperative course was uneventful apart from a mild biological postoperative hypocalcemia that was observed in all cases. The management of this hypocalcemia required supplementation with oral calcium with good evolution. No surgical morbidity was observed after a mean follow-up of 7.9 months (range of 5–18 months).

**Table 1:** General characteristics of our patients (PTH =Para Thyroid Hormone, PA= Parathyroid Adenoma).

Nº	Age/Sex	Clinical presentation	Biochemistry	Neck ultrasound	Thoracic CT-scan/MRI	Sestamibi-MIBI scan	Surgical approach	Tumor location	Postoperative course	Hospital stay
1	61/female	Osteoporosis with spontaneous spinal fracture (D10-11) and forearm fracture (all the 2 bones)	PTH = 10 N Ca = 142 mg/l	Benign thyroid nodule	CT-scan = hypervascular lesion of the middle and superior mesiastinum	Laterotracheal parathyroid adenoma (PA)	Cervicotomy	Laterotracheal	Hypocalcemia, normalised after 2 weeks	**10 days**
2	44/Female	Reccurrent kidney stones	PTH = 4 N Ca = 114 mg/l	Thyroid goiter	CT-scan = spontaneously hyperdense anterior mediastinal tissular lesion	Anterior mediastinal PA	Manubriotomy	Right thymic lobe	Hypocalcemia, normalisation after 7 days	**11 days**
3	54/Female	Malignant hypercalcemia Gallstones	PTH = 6 N Ca = 131 mg/l		MRI = superior mediastinal lesion with T1 hyposignal		Cervico-Manubriotomy	Interjugulocarotidian with contact with subclavicular artery	Uneventful	**8 days**
4	70/Female	Hypercalcemia	PTH = 3 N Ca = 138 mg/l		CT-scan = tissular retrotracheal lesion of the posterior mediastinum	Posterior mediastinal PA	Right posterolateral thoracotomy	Retrotracheal with tracheal contact	Hypocalcemia with clinical symptoms, normalisation in 5 days	**6 days**
5	55/Female	Kidney stones	PTH = 749 pg/ml Ca = 126 mg/l		CT-scan = hyperdense antero-superior mediastinal tissular lesion	–	Median total sternotomy	Intrathymic	Hypocalcemia, normalisation in 12 days	**13 days**
6	68/Female	Bone pain with spontaneous femoral fracture	PTH = 453 pg/ml Ca = 110 mg/l		CT-scan = antero-superior mediastinal lesion with contact with superior vena cava (SVC)	Anterior mediastinal PA	Manubriotomy	Thymic lodge	Hypocalcemia with clinical symptoms, normalisation in 4 days	**7 days**
7	62/Female	Pathologic diaphyseal fracture of the left femur	PTH = 5,6 N Ca = 127mg/l		CT-scan = anterior mediastinal tissular lesion	Anterior mediastinal PA	Cervico-Manubriotomy	Between brachiocephalic artery and SVC	Hypocalcemia, normalisation in 3 days	**8 days**

## 4 Discussion

The ectopic parathyroid adenomas have a variable incidence according to many reported studies [4,5]. Phitayakorn and Mc Henry, in a series of 231 patients operated for hyperparathyroidism, had reported 16% of ectopic lesions among which 3% were intra-thymic [6]. In fact the caudal parathyroid glands and the thymus embryologically derived from the third branchial pouch before their respective migration in the cervical and mediastinal level [7]. This migration may explain the possibility of supernumerary parathyroid and ectopic, especially in the chest cavity. The diagnosis is usually suspected in primary hyperparathyroidism. In general, symptoms are characterized by recurrent nephrolithiasis, signs related to phosphocalcic metabolic disorders, muscle weakness, heart rhythm disorders or spontaneous bone fractures as in some cases of our current series [8]. Thus in the presence of clinical signs associated with phosphocalcic metabolic disorders, the clinical and radiological examination of the neck should search for the possibility of a cervical mass in relation with a parathyroid adenoma. Unfortunately for the majority of patients, these radiological investigations are not contributory. In the specific case of our patients, the persistence of clinical and biological signs associated with hypercalcemia, contrast CT scan allowed us to identify the lesion in the mediastinum in 6 patients. Thus, to support the diagnosis, scintigraphy with technetium 99 showed intense uptake of the radiotracer in the mediastinal mass in 5 patients. Indeed, the sestamibi-scan is currently considered the technique of choice since it has a sensitivity of approximately 95% and provides better picture quality than CT-scan with a lower radiation [9,10]. Mitchell has reported in his review 16 adenomas among 17 cases and 19 of 21 cases of glandular hyperplasia [11]. In the specific case of our patients scintigraphy allowed to clearly specify the injury thus avoiding the realization of unnecessary cervicotomy. According to Rousseau et al. [1], in the presence of a parathyroid adenoma, surgical resection of hyperfunctioning tissue is the gold standard. The current trend is to offer surgery to all patients in whom the diagnosis of primary hyperparathyroidism is made, which includes asymptomatic patients, regardless of age [1,12,13]. Indeed, several studies have shown adverse effects of hyperparathyroidism: increased risk of premature death, hypertension, myocardial hypertrophy, bone loss particularly in elderly women [1,[14], [15], [16]]. In symptomatic patients, parathyroidectomy normalizes biochemistry and increase the bone density [1,17]. Surgical procedures are still controversial. Sternotomy or thoracotomy are the first classical approaches for ectopic parathyroid adenomas of mediastinal location as reported by some authors [1,18]. In 1994, an alternative video-assisted thoracoscopy was proposed in order to reduce the extension of the first classical surgical approaches and decrease its morbidity [1,19,20]. This video-assisted approach allows good visualization of the lesion in the mediastinum. Its disadvantages are the lack of vision in 3D dimension and a loss of dexterity of the surgeon in comparison to conventional surgery [1,21]. Whatever the intended surgical technique, preoperative localization of the lesion is often difficult despite technical investigations that are now becoming more sophisticated. In order to minimize the risk of leaving a hyperfunctioning tissue, and therefore reoperation, our team has initiated an intraoperative parathyroid hormone assay. The goal is to find a parathyroid hormone rate of decrease of about 50%. This is an old technique but still valuable in our context as no case of recurrence was noted in our series. However the reduced sample size limits the sensitivity and specificity of this technique, this is in accordance with Najafian A et al. findings [22]. In case of surgical management, hypocalcemia is a constant postoperative complication. According to Rousseau et al. [1] its absence makes questionnable the complete resection of the tumoral tissue. The signs most commonly found are clinical neuromuscular excitability with paresthesia, cramps and/or tetany. At a more advanced stage there is impaired consciousness, seizures and laryngospasm or bronchospasm [1,23]. It is a general rule in patients operated for primary hyperparathyroidism. It should only be treated if it becomes symptomatic because serum calcium levels returns to normal levels on the 4th or 5th day post operatively [1]. Below 1.9 mmol/L associated with muscle symptoms or electrocardiographic abnormality, calcium infusions are administered over 24–48 hours [1]. Persistent hypocalcemia beyond this delay is usually due to an uptake of calcium and phosphorus by the bones requiring supplementation and vitamin D therapy [1,24].

## 5 Conclusion

The ectopic parathyroid adenomas are rare and their mediastinal location in varying sites makes challenging their management. The morphological assessment including CT-scan coupled with scintigraphy are crucial to localize most of the parathyroid ectopias. Despite the performance of these increasingly sophisticated investigative techniques, the surgeon must perform an accurate and meticulous dissection to minimize the risk of leaving behind a hyperfunctioning parathyroid tissue. The intraoperative monitoring of parathyroid hormone is of great help, to guide the surgeon in his excisional act.

## Ethical approval

There is no ethical committee in our country (Not applicable for this manuscript).

## Sources of funding

There are no sources of funding

## Authors’ contributions

All authors contributed to the study design. SR, HH and ML performed data collection and data analysis. SR, EB and HH performed the cost analysis. All authors critically interpreted all data analysis. YO and MS composed the manuscript, and all the remaining authors provided critical edits to the final draft. All authors read and approved the final manuscript.

## Conflicts of interest

There are no conflicts of interest.

## Trial registry number

Researchregistry4677.

## Guarantor

Sani Rabiou.

## Conflicts of interest

The authors declare that they have no competing interests.

## Availability of data and materials

The datasets used and/or analyzed during the current study available from the corresponding author on reasonable request.

## Consent for publication

Not applicable.

## Provenance and peer review

Not commissioned, externally peer reviewed.

## Appendix A Supplementary data

DataProfile DataProfile

process_2018_table process_2018_table
